# The Use of Black Cohosh during Pregnancy

**Published:** 1873-09

**Authors:** Ely Van DeWarker

**Affiliations:** Syracuse, N. Y.


### THE USE OF BLACK COHOSH DURING PREGNANCY.

BY ELY VAN DEWARKER, M.D., SYRACUSE, N. Y.

On page 230 of the April number of the RECORD, there is an extract from the *Eclectic Medical Journal*, relating to the medical properties of this plant. One sentence in that extract calls, I think, for a statement of my experience of its therapeutics. The sentence reads: "It is said that it has been given to gravid females for the removal of cough, with the effect of producing a speedy abortion." This is so contrary to my experience of its action—and I confine its use almost exclusively to the pregnant condition—that I must protest against the plant being given a bad name. I cannot help regarding a good remedy in the light of a friend, and in that relation I feel bound to defend its character.

Women are oftentimes the subjects of distressing symptoms as pregnancy advances. Among these are a train of nervous symptoms as pregnancy advances. Restlessness, sleeplessless, darting pains in the back, flanks and thighs, and stiffness and soreness in movement, are very common and troublesome. For these conditions I find black cohosh a sovereign remedy. I give thirty minims, or half a teaspoonful, of the flud extract at bed-time, in cases of restlessness, and in case of neuralgia of the lumbar or abdominal muscles, or in cases of stiffness or soreness in movement, the extract may be given in the same amount, at intervals of three to five hours during the day.

I know of no medicine equal to the black cohosh for the conditions mentioned above. I have used the plant for these cases during the last ten years, and I never saw an abortion produced. I am aware of the drug having been used as a parturifacient, and with good results, I am told; but as I regard the action a class of drugs, when so used—and cohort among the number—that is not conflicting in any way with the fact that these same drugs may be safely employed during gestation. Quinine has a reputation well established as a parturifacient, but none of us would hesitate to use it, I believe, when called for in the pregnant female. I place ergot in the same class. I have explained, elsewhere, my theory of this.*

*Detection of Criminal Abortion, and a Study of Fœticidal Drugs, p. 47, 800, Boston, James Campbell.

This theory may be formulated. Uterine contractions, or irritability, being once established, various substances, when introduced into the system, are capable of promoting or intensifying such contractions or irritability, which, when the uterine nerves and muscles are quiescent, have no such power. Quinine, ergot and actea racemosa I class among these agents which so act. I know of no other way of explaining phenomena, which, at a superficial glance, appears contradictory.

I deny to any of these drugs the power of inducing primary uterine contractions *per se.* Acting upon this theory, I have used these agents during gestation with perfect safety to the uterine contents. So far as black cohosh is concerned, the reader may use it during pregnancy, when indicated, without any fear that it will prove an ecbolic.

---

Gelsemium in Gynæcology.—Dr. H. V. Ferrell, Carbondale, Illinois, writes to *Clinic* that two doses of gelsemium, of ten drops each, entirely relieved the excessive irratability of the uterus in a case of endometritis, and the os, which was, before the administration of gelsemium, rigid and contracted, dilated. Another case—suppression of lochia, and retention of part of the placenta—the result of the stupidity of a midwife. Present condition of the patient alarming; in which, on examination, the os was found firmly contracted, and hardly admitted the end of the index finger. The patient was treated with gelsemium and belladonna, and in twelve hours the os dilated so as to admit two fingers, and permit the extraction of the retained placenta, etc., when the patient recovered without further trouble.

Gelsemium (in five drops every hour, in water, till relief is obtained, and then at longer intervals) has relieved after-pains in very severe cases. Dr. Shaw, of Guthrie, Iowa, claims to have had the best results from gelsemium in the treatment of uterine leucorrhœa, and Dr. Mobsworth, of DesMoines, places more reliance in it for the successful treatment of chronic dysmenorrhœa than in any other remedy. Both these gentlemen say its good effects in these affections are frequently as prompt and decided as is quinia in intermittents.—*St. Louis Medical Archives.*

